# Use of artificial intelligence in analysis of endoscopic images to detect residual disease or regrowth in rectal patients with complete clinical response to neoadjuvant chemoradiotherapy

**DOI:** 10.1007/s10151-026-03333-5

**Published:** 2026-05-14

**Authors:** M. A. Javed, M. Mascarenhas, F. Mendes, E. Carvalho, R. Rajan, A. Santos, Z. Khan, I. Blake, S. Ahmed

**Affiliations:** 1 Institute of Systems Molecular and Integrated Biology, The University of Liverpool, Liverpool, UK; 2 Precision Medicine Unit, Department of Gastroenterology, Unidade Local de Saúde São João, Porto, Portugal; 3 WGO Gastroenterology and Hepatology Training Center, Porto, Portugal; 4 Faculty of Medicine, University of Porto, Porto, Portugal; 5 CINTESIS-RISE, Department of Community Medicine, Information and Health Decision Sciences (MEDCIDS), Faculty of Medicine, University of Porto, Porto, Portugal; 6 DigestAID, Digestive Artificial Intelligence Development, Porto, Portugal; 7 INEGI, Instituto de Ciência E Inovação Em Engenharia Mecânica E Engenharia Industrial, Porto, Portugal; 8 Department of Colorectal Surgery, Liverpool University Hospital NHS Foundation Trust, Liverpool, UK

**Keywords:** Rectal cancer, Complete clinical response, Watch and Wait and artificial intelligence

## Abstract

**Background:**

Patients with rectal cancer who have a complete clinical response (cCR) to neoadjuvant chemo/radiotherapy (nCRT) may opt for organ preservation and watch and wait (W&W). This consists of an intense surveillance program including serial endoscopies, pelvic magnetic resonance imaging (MRI), carcinoembryonic antigen (CEA), and computed tomography (CT) scans to detect regrowth at an early stage. However, residual lesions or regrowths can be challenging to identify endoscopically, due to mucosal changes such as friability and neovascularization. We developed a novel deep learning model to assist in the detection of residual or regrown rectal cancer lesions during proctosigmoidoscopy.

**Methods:**

We trained a convolutional neural network (Wide ResNet-101-2) on a dataset of 1795 annotated frames from proctosigmoidoscopy exams of 97 patients treated at a tertiary referral center. Residual or regrowth was defined by histopathological confirmation. The dataset was split into training and testing cohorts using a 90/10% patient-level split.

**Results:**

Out of 97 patients, 24 (363 frames) had confirmed residual disease or regrowths, while 73 (1432 frames) presented normal rectal mucosa. The model achieved an overall accuracy of 92.8%, with a sensitivity of 80.0%, specificity of 97.3%, positive predictive value (PPV) of 90.9%, negative predictive value (NPV) of 94.0%, and an area under the receiver operating characteristic curve (AUROC) of 0.886.

**Conclusions:**

To the best of our knowledge, this is the first deep learning model specifically developed for the detection of residual disease or regrowth following cCR in W&W patients during endoscopic examination. This tool has the potential to aid lesion detection, guide clinical decision-making, and increase opportunities for salvage, curative treatment strategies.

## Introduction

Colorectal cancer (CRC) is the third most frequently diagnosed cancer globally and the second leading cause of cancer-related mortality. Rectal cancer comprises about 30–35% of all CRC cases, representing a major portion of the global disease impact [1]. Management of locally advanced rectal cancer presents considerable clinical challenges and typically necessitates multimodal therapeutic approaches to optimize patient outcomes. Evidence demonstrates that neoadjuvant chemo/radiotherapy (nCRT) substantially improves local disease control and overall survival, establishing it as a fundamental component of contemporary rectal cancer treatment protocols [2, 3]. Furthermore, total neoadjuvant therapy (TNT) and contact radiotherapy in the form of papillon are being considered as alternatives to surgery for management of rectal cancer in selected cases. A key therapeutic milestone following neoadjuvant treatment is the attainment of a complete clinical response (cCR), characterized by the absence of detectable malignancy on the basis of clinical, radiological, and endoscopic assessments [4].

The emergence of the cCR subgroup has led to a paradigm shift in rectal cancer management, enabling the adoption of organ-preserving “watch and wait” (W&W) strategies. This approach circumvents the morbidity and functional deficits associated with total mesorectal excision. Patients in this cohort are closely monitored through rigorous follow-up involving serial endoscopic examinations, magnetic resonance imaging (MRI), computed tomography (CT), and CEA levels [5]. While this strategy enhances quality of life and mitigates surgical risks, it carries the inherent risk that approximately 20–25% of patients with initial cCR will develop luminal regrowths, predominantly within the first 2 years. Therefore, early and accurate detection of residual disease or regrowths during surveillance is critical to ensure oncologic safety and facilitate timely salvage interventions.

Despite regular follow-up, endoscopic detection of regrowths remains challenging. Radiation-induced mucosal alterations including fibrosis, friability, and neovascularization can obscure visualization and simulate recurrent lesions, resulting in diagnostic ambiguity. These factors contribute to substantial interobserver variability among endoscopists, potentially causing both over- and underdiagnosis. Given these diagnostic challenges, there is a pressing need for objective, reliable, and reproducible tools to enhance detection accuracy in post-nCRT surveillance.

Artificial intelligence (AI), particularly deep learning (DL), has recently shown significant promise in advancing medical image analysis. Among DL architectures, convolutional neural networks (CNNs) have proven especially proficient in interpreting visual data such as endoscopic and radiologic images. Within gastroenterology, CNN-based algorithms have been successfully applied to detect colonic polyps and to distinguish neoplastic from non-neoplastic lesions with notable accuracy [6].

Despite these advances, the utilization of AI in the post nCRT endoscopic surveillance of rectal cancer remains insufficiently explored. Very few studies have focused on developing AI-driven models to detect residual disease or regrowth in patients exhibiting cCR, representing a notable gap in oncologic care [7]. AI’s capability to detect subtle mucosal architectural changes often overlooked by human observers could markedly improve early detection of regrowths, thereby minimizing the need for invasive biopsies or additional imaging. Moreover, AI tools can provide consistent, scalable support as adjunct readers, augmenting clinical decision-making without supplanting expert judgment. The aim of this study is to develop a novel AI model for analysis of endoscopic images following cCR in rectal cancer.

## Methods

### Study design

This retrospective, observational study was conducted at University Hospitals Liverpool and affiliated Clatterbridge Cancer Centre, tertiary referral centers specializing in the management of patients with rectal cancer. Study population included individuals who were on W&W protocol following cCR to neoadjuvant chemo/radiotherapy; normal rectum examinations were included as controls. Eligibility criteria for rectal cancer inclusion were aligned with standards established in previous multicenter studies, including those by Shen et al., to maintain methodological consistency [8]. Inclusion criteria required patients with rectal cancer to be over 18 years of age, have histologically confirmed rectal adenocarcinoma, have completed neoadjuvant chemo/radiotherapy, and exhibit a cCR as determined by colorectal multidisciplinary team (MDT) on MRI and endoscopy at least 6 weeks after completion of neoadjuvant treatment. We follow the international consensus recommendations of four monthly endoscopy in the first 2 years, followed by six monthly endoscopic examinations in last 3 years of surveillance [5]. Patients classified as cCR, who were referred from peripheral hospitals and had a repeat scope at tertiary center who were found to have malignancy, were classified as “residual disease.” All patients who were diagnosed after index surveillance endoscopy were classified as “regrowth.” Furthermore, availability of high-resolution post-nCRT endoscopic images suitable for deep learning analysis was mandatory for inclusion. For each patient, images from initial assessment and subsequent endoscopic follow-up examinations were included.

**Fig. 1:**
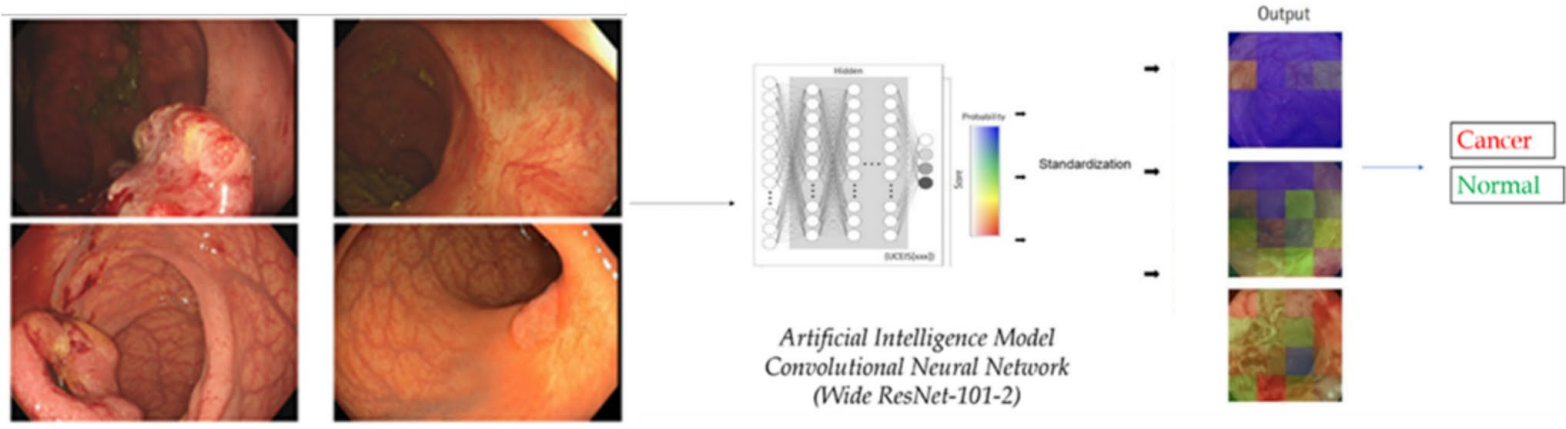
Visual pipeline of endoscopic images processed through a CNN model. Representative endoscopic frames analyzed by the AI model, illustrating pre- and post-treatment appearances. The system correctly identified complete response (top right) and residual disease (bottom right)

Figure 1 presents a visual pipeline in which endoscopic images of rectal cancer are processed through a convolutional neural network (CNN) model. The system is designed to automatically exclude suboptimal or inadequate frames, ensuring only high-quality inputs are analyzed. The trained AI model then classifies the remaining frames to support clinical decision-making by distinguishing between post-treatment fibrotic scarring and true tumor regrowth, thereby enhancing diagnostic accuracy in the surveillance of patients following neoadjuvant therapy.

### Image acquisition and preprocessing

High-resolution white-light proctosigmoidoscopy was employed and regions of interest (ROI) were selected on the basis of optimal mucosal visualization, excluding frames compromised by artifacts such as bleeding, motion blur, or poor focus. A standardized preprocessing pipeline was implemented to enhance image quality and consistency across the dataset. This included histogram equalization to normalize lighting, spatial rescaling to ensure uniform resolution, and the application of denoising filters to reduce background noise. Distinct from studies primarily adapting techniques from natural image processing, our methodology also incorporated targeted artifact removal and image stabilization procedures to further improve image fidelity for deep learning analysis.

### Data annotation and reference standard

Image annotations were validated against histopathological findings, serving as the diagnostic gold standard. Frames corresponding to confirmed residual disease or regrowths were categorized as “tumor present,” while those obtained from healthy rectums and patients who maintained a cCR without evidence of recurrence for a minimum follow-up period of 24 months were labeled as “no tumor.” This annotation strategy is consistent with the classification framework utilized in other CNNs [6]. The annotated endoscopic image dataset was used for training and optimizing the deep-learning-based AI model at University of Porto.

### Deep learning architecture

The model architecture was built upon the Wide ResNet-101-2 framework, a deep residual network variant optimized for extracting complex features from high-dimensional medical imaging data. Initial pretraining on the ImageNet dataset enabled effective transfer learning, facilitating improved convergence and performance on the relatively limited endoscopic dataset, an approach consistent with established practices in AI-based endoscopic analysis [9]. To enhance generalizability and reduce overfitting, the network incorporated batch normalization and dropout layers. Additionally, spatial attention mechanisms were integrated to focus on critical mucosal textures and vascular patterns relevant to the detection of residual or recurrent disease.

The dataset was split at the subject level using a 90/10 ratio, allocating 87 patients (1615 frames) to the training set and 10 patients (180 frames) to the test set (Table 1). To prevent data leakage, patient-level separation was ensured, an approach consistent with the methodology used by [7]. To enhance model generalizability, image augmentation techniques such as rotation, flipping, and brightness adjustment were applied, in line with practices adopted in multicenter endoscopic research.

**Table 1:** Key components of the deep learning model architecture and training parameters used in the study

Component	Description
Model architecture	Wide ResNet-101-2
Input image dimensions	224 × 224 pixels (resized)
Loss function	Categorical cross-entropy
Regularization	Dropout (rate = 0.3), batch normalization
Data augmentation	Rotation, horizontal/vertical flip, brightness/contrast shift
Epochs	50 (with early stopping if no improvement in 10 consecutive validation epochs)
Training–validation–test split	90/10% patient level split

The Wide ResNet-101–2 model was trained on resized endoscopic images (224 × 224 pixels) using categorical cross-entropy loss. Regularization techniques included dropout and batch normalization. Data augmentation strategies were applied to enhance generalization. A patient-level 90:10 training and validation split was used, with early stopping triggered after ten consecutive validation epochs without improvement.

### Testing and performance evaluation

The evaluation framework incorporated key performance metrics including sensitivity, specificity, accuracy, positive predictive value (PPV), negative predictive value (NPV), and area under the receiver operating characteristic curve (AUROC), with 95% confidence intervals estimated for AUROC. Confusion matrix analysis was conducted to assess misclassification rates, and statistical reliability was ensured using binomial exact tests [10].

### Statistical analysis

Descriptive statistics summarized the patient count, frame distribution per class, and the ratio of normal to abnormal cases. Primary classification metrics such as accuracy, sensitivity (recall), specificity, PPV, NPV, and AUROC were computed at the frame level by comparing model outputs against expert-annotated ground truth.

To ensure statistical reliability, 95% confidence intervals (CIs) were estimated using a nonparametric bootstrapping approach with 1000 iterations. A confusion matrix was constructed to visualize true positives (TP), false positives (FP), true negatives (TN), and false negatives (FN), serving as the basis for calculating sensitivity, specificity, PPV and NPV.

The statistical significance of the association between actual and predicted classifications was assessed using a chi-squared test of independence, with a two-tailed *p*-value < 0.05 considered significant. Additionally, the receiver operating characteristic (ROC) curve was plotted, and the AUROC was computed to evaluate the model’s discriminative capacity across varying thresholds. AUROC values between 0.8 and 0.9 were interpreted as indicative of excellent diagnostic performance. All analyses were performed using Python (v3.9).

## Results

### Cohort overview and image data distribution

Table 2 summarizes baseline characteristics of study population. Between January 2020 and December 2024, 97 patients were included, of whom 66 were initially classified to have a cCR after neoadjuvant chemo/radiotherapy. From these, 1795 annotated frames were collected. A total of 24 patients (363 frames) had histologically confirmed regrowths, while 73 patients (1432 frames) exhibited normal rectal mucosa or complete response.

**Table 2:** Patient demographics and tumor characteristics of study population

	Controls *n* = 31	Disease *n* = 24	Complete response *n* = 42
Age (years), median	61	66.5	69
Gender			
Male (number)	17	14	22
Female (number)	14	10	20
Stage (number)			
I	N/A	5	12
II		6	9
II		13	18
IV		0	3
Location (number)			
Upper	N/A	0	5
Middle		10	14
Low		14	23
Treatment (number)			
SCRT	N/A	4	7
SCRT + papillon		2	2
LCCRT		11	23
LCCRT + papillon		3	1
TNT		4	8
Papillon		0	1
Time to recurrence (months), median	N/A	21	N/A

*IQR* interquartile range, *SCRT* short-course radiotherapy, *LCCRT* long-course chemoradiotherapy, *TNT* total neoadjuvant therapy

### Model performance metrics

**Fig. 2:**
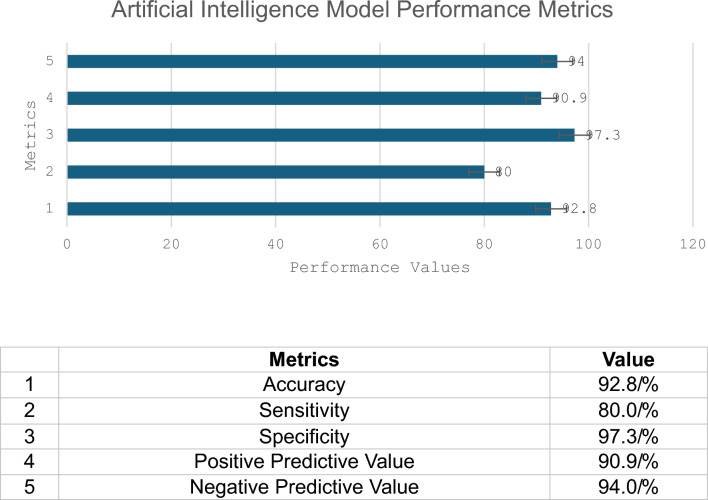
Diagnostic performance metrics of the AI model, including accuracy, sensitivity, specificity, predictive values, and AUROC with 95% confidence interval

Figure 2 demonstrates the model’s ability to differentiate between normal mucosa and regrowth with high overall precision.

**Fig. 3:**
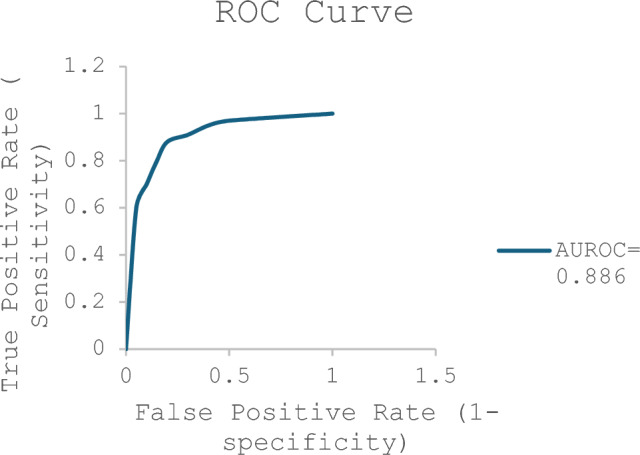
Receiver operating characteristic curve of the AI model denotes 95% confidence intervals

The model achieved an accuracy of 92.8%, indicating that approximately 1667 out of 1795 frames were correctly classified. Sensitivity was 80%, indicating the model accurately flagged four out of every five disease frames, and specificity was 97.3% (Fig. 3).

The current model is calculated to have a positive predictive value (PPV) of 90.9%, negative predictive value (NPV) of 94.0% and the area under the receiver operating characteristic curve (AUROC) was 0.886, reflecting excellent discriminative performance.

### Confusion matrix and classification breakdown

**Fig. 4:**
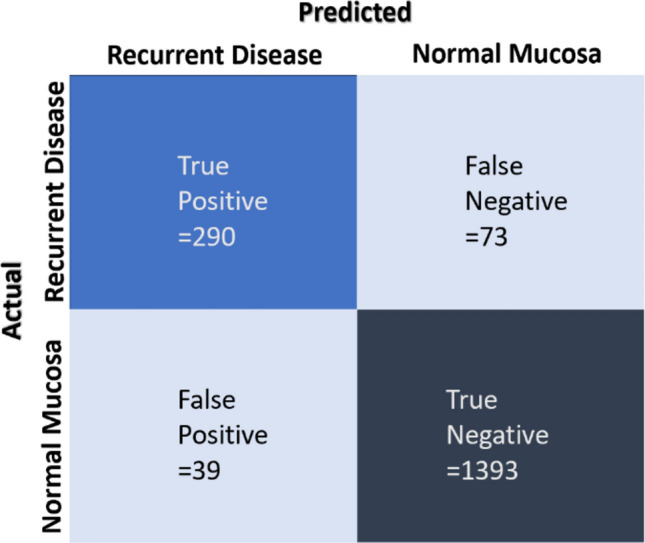
Confusion matrix illustrating the AI model’s performance in classifying endoscopic frames. The model accurately identified cases of residual or recurrent disease (true positives) and normal mucosa consistent with complete clinical response (true negatives), with few misclassifications, reflecting strong specificity and overall diagnostic reliability

The confusion matrix analysis further elaborates the model’s performance at the frame level, shown in Fig. 4.

The AI model accurately detected 290 frames containing residual disease or regrowths (true positives), reflecting its effectiveness in identifying cases requiring clinical intervention. However, 73 disease frames were misclassified as normal (false negatives), indicating some limitations in sensitivity. Additionally, the model misclassified 39 frames of normal mucosa as diseased (false positives). Notably, it correctly identified 1393 frames of normal mucosa (true negatives), highlighting its high specificity and reliability in distinguishing healthy tissue. [11].

### Statistical significance and confidence intervals

To assess the robustness of the AI model, 95% confidence intervals (CIs) were calculated for the primary performance metrics using bootstrapping techniques. The model achieved an accuracy of 92.8% (95% CI 91.4–94.1%), sensitivity of 80.0% (95% CI 75.2–84.6%), specificity of 97.3% (95% CI 96.1–98.4%), positive predictive value (PPV) of 90.9% (95% CI 87.2–94.6%), negative predictive value (NPV) of 94.0% (95% CI 92.5–95.5%), and an area under the receiver operating characteristic curve (AUROC) of 0.886 (95% CI 0.863–0.910). Furthermore, chi-squared testing confirmed significant class discrimination ability (*p* < 0.001), validating that the model’s predictive power is statistically significant and exceeds random classification.

## Discussion

The performance metrics from our model indicate that the proposed AI framework holds significant potential as a supportive diagnostic tool during endoscopic surveillance for patients with rectal cancer on W&W pathway. Timely and accurate identification of residual disease or lesions that have regrown is crucial for initiating appropriate salvage treatments while avoiding unnecessary surgical procedures when no disease is present. The model’s high NPV of 94.0% is particularly meaningful in the context of watch-and-wait strategies, as it suggests that frames predicted as normal are indeed likely to be disease-free. This reduces the need for additional investigations, thereby minimizing patient and financial burden. Conversely, the PPV of 90.9% provides reassurance that when the AI flags a lesion as suspicious, it is very likely to be a true positive. This enhances clinical confidence in leveraging the AI model to support decision-making, particularly in borderline cases where visual assessments may be subjective or unclear. Moreover, the high specificity of 97.3% highlights the model’s strong ability to avoid false positives, which are common in traditional evaluations due to post-treatment changes such as inflammation, mucosal irregularities, or scarring. This further reinforces the AI tool’s utility in improving diagnostic precision in endoscopic surveillance.

Our model’s higher accuracy (92.8%) and AUROC (0.886) indicate more precise discrimination of recurrence within a single-center dataset. The current model’s performance sits between these extremes, suggesting strong internal validity with potential for broader generalizability.

Previous studies have reported mixed outcomes in detecting rectal cancer regrowth following nCRT using conventional imaging techniques. Studies report considerable interobserver variability in post-treatment assessments and limitations of MRI in accurately distinguishing post-treatment scarring from residual tumor tissue [12]. In contrast, the AI model developed in the present study demonstrated enhanced performance, particularly in interpreting endoscopic images, achieving greater accuracy and fewer false positives compared with conventional approaches [13–16], as summarized in Table 3.

**Table 3:** Benchmark comparison with published AI studies

Study and year	Method	Modality	AUROC	Accuracy	Sensitivity	Specificity
Esteva et al. [17] (2017)	Deep CNN	Dermoscopy	0.911	89.5%	76.0%	94.0%
Smith et al. [18] (2021)	DL endoscopy	Anal neoplasia	0.793	85.1%	70.0%	90.2%
Zhang et al. [19] (2021)	AI—Colonoscopy	CRC detection	0.831	88.3%	75.2%	91.8%
Current study (2025)	Wide ResNet-101-2	Proctosigmoidoscopy	**0.886**	**92.8%**	**80.0%**	**97.3%**

When compared with existing AI-based approaches for endoscopic surveillance in colorectal and rectal cancer, the current model shows clear improvements. Previous studies have reported variable sensitivity (70–90%) and specificity (80–95%) across models. In contrast, the present study’s AUROC of 0.886, along with superior PPV and specificity, indicates stronger performance in detecting subtle post-nCRT mucosal abnormalities. These advancements may be attributed to the use of the WideResNet-101–2 architecture, which is well suited for extracting deep, abstract features in complex post-treatment imaging data.

Despite encouraging results, the study has several limitations. Given the retrospective nature of the study, it was not possible to quantify the level of confidence the endoscopist had for lesions to be malignant, which could be compared with AI and histological diagnosis. Including patients with 2-year follow-up for cCR may be a limitation, as local regrowth can still occur later, although less frequently. The dataset, while enriched, originates from a single tertiary center, potentially limiting external applicability. Additionally, the relatively small number of recurrence-positive cases may restrict the model’s generalizability to broader and more diverse populations. The presence of 48 false negatives also presents a concern, emphasizing the importance of integrating additional diagnostic modalities, such as MRI and CEA levels, to support final clinical decisions. Analysis of false positives revealed that many incorrectly classified normal frames exhibited features such as scarring or radiation-induced mucosal changes, which closely resemble early recurrence. This underscores the need for incorporating complementary diagnostic modalities, such as MRI or histopathology, to improve diagnostic accuracy and confidence.

Ongoing work focuses on incorporating temporal data from serial endoscopic exams to improve the model’s predictive consistency over time and include data on endoscopic degree of confidence in interpreting a lesion as malignant. Moreover, the inclusion of multimodal inputs, such as radiomics, serum biomarkers, and MRI findings, is expected to enhance diagnostic accuracy beyond that of image-only system [20]. Additionally, despite strong overall accuracy and AUROC, the model’s generalizability across different clinical settings remains unproven. Variations in endoscopic equipment, lighting, mucosal staining techniques, and patient demographics could impact performance. Therefore, prospective validation across multiple centers is essential to confirm broader applicability.

By delivering standardized, reproducible assessments, the model has the potential to improve the efficiency of endoscopic surveillance and reduce clinician workload. Early detection of regrowth supports timely therapeutic action, potentially improving clinical outcomes. In addition, widespread adoption of such technology may reduce healthcare expenditures by limiting unnecessary investigations. Collectively, this AI model offers a meaningful improvement to existing W&W surveillance protocols, supporting more effective and patient-centered management.

The W&W approach, which emphasizes organ preservation in patients with cCR, depends on accurate and reproducible detection of regrowth. Studies have reported accuracy ranging from 53% to 90% and significant variability between observers using traditional assessment methods [21]. The current CNN model’s combination of high specificity and acceptable sensitivity may provide a more standardized and dependable alternative, contributing to safer and more consistent surveillance workflows.

The choice of the Wide ResNet-101–2 architecture, characterized by increased width rather than depth, facilitates deeper feature extraction and mitigates vanishing gradient issues. This architecture is particularly well suited for capturing complex mucosal and vascular patterns in endoscopic imagery, offering potential advantages over alternative frameworks such as EfficientNet [22] or DenseNet [23].

For effective clinical translation, future integration of the model into endoscopic systems with real-time inference capabilities, saliency map visualization, and user-friendly interfaces will be essential. The most effective deployment strategy involves augmenting, rather than replacing, the clinician’s role. Future research should include prospective video-based validation studies and the incorporation of clinician feedback to facilitate clinical acceptance and workflow integration.

On the contrary, despite satisfactory accuracy, the black-box nature of deep learning models reduces clinician confidence in model decisions, thereby decreasing their applicability. In fact, the development of models with increased complexity is often associated with a decreased clinician understanding of the reasons behind such decisions [24]. In this context, explainable AI (XAI) aims to address this limitation by providing specific tools to help clinicians understand the reasons behind model outputs. Future studies will therefore focus on developing XAI tools for detecting specific regions of an image that may indicate residual lesions or recurrence. As validated in previous studies, the use of heatmaps or bounding boxes would be of great interest [25]. These mechanisms would not only increase confidence in model decisions, but also potentially improve the selection of biopsy sites, ultimately transforming patient outcomes.

In conclusion, this tool has the potential to facilitate endoscopic diagnosis of residual disease or regrowths in patients with rectal cancer who opt for organ preservation following cCR to neoadjuvant treatment, guide clinical decision-making, and increase opportunities for salvage, curative treatment strategies. To strengthen future deployment, efforts should focus on multicenter external validation, integration of multimodal inputs (e.g., MRI, serum biomarkers), and prospective clinical trials comparing standard care with AI-augmented workflows. Emphasis on explainability, through tools such as attention or saliency maps, as well as hardware optimization and regulatory compliance, will be vital to achieving safe and effective implementation in clinical practice [26].

## Data Availability

Additional data are available upon reasonable request.
